# Modeling Treatment Response in Tuberculosis Early Bactericidal Activity Trials

**DOI:** 10.1002/psp4.70311

**Published:** 2026-08-07

**Authors:** Mairi C. W. McClean, Simon E. Koele, Julia Dreisbach, Susanne Mirold‐Mei, Fred Njeleka, Daniel Mapamba, Bariki Mtafya, Patrick P. J. Phillips, Veronique R. De Jager, Rodney Dawson, Kim Narunsky, Andreas H. Diacon, Elin M. Svensson, Norbert Heinrich, Francesco Paolo Casale, Michael Hoelscher

**Affiliations:** ^1^ Institute of Infectious Diseases and Tropical Medicine, LMU University Hospital Munich Germany; ^2^ Institute of AI for Health, Helmholtz Center Munich—German Research Center for Environmental Health Neuherberg Germany; ^3^ Institute for Medical Information Processing, Biometry, and Epidemiology (IBE), Pettenkofer School of Public Health LMU Medizin, Ludwig‐Maximilians‐Universität München Munich Germany; ^4^ Helmholtz Pioneer Campus Helmholtz Zentrum München—German Research Center for Environmental Health Neuherberg Germany; ^5^ Department of Pharmacy, Pharmacology, and Toxicology Radboud Institute for Medical Innovation, Radboud University Medical Center Nijmegen the Netherlands; ^6^ German Center for Infection Research (DZIF), Munich Partner Site Munich Germany; ^7^ National Institute for Medical Research‐Mbeya Medical Research Center Mbeya Tanzania; ^8^ UCSF Center for Tuberculosis University of California San Francisco San Francisco California USA; ^9^ TASK Applied Science Clinical Research Center Cape Town South Africa; ^10^ Department of Medicine University of Cape Town Lung Institute Cape Town South Africa; ^11^ Department of Pharmacy Uppsala University Uppsala Sweden; ^12^ Fraunhofer Institute for Translational Medicine and Pharmacology ITMP, Immunology, Infection, and Pandemic Research Munich Germany; ^13^ School of Computation, Information and Technology Technical University of Munich Garching Germany; ^14^ Unit Global Health, Helmholtz Zentrum München—German Research Centre for Environmental Health Neuherberg Germany

**Keywords:** biomarkers, BTZ‐043, EBA study, machine learning, tuberculosis

## Abstract

Culture‐based monitoring of bacterial load is slow and susceptible to missing data, contributing to the length and cost of TB clinical trials. Non‐culture‐based alternatives, like the Tuberculosis Molecular Load Bacterial Assay (TB‐MBLA), could represent a solution. Our objectives were to evaluate TB‐MBLA as a biomarker in early bactericidal activity (EBA) studies and explore whether combining biomarkers with joint modeling could provide insight into underlying biological processes. We generated TB‐MBLA (LifeArc) data from sputum samples from all 78 patients from the PanACEA BTZ‐043 Phase Ib/IIa trial and derived a summary measure of the joint distribution of the three TB‐MBLA, colony forming units (CFU), and time‐to‐positivity (TTP) biomarkers as the first principal component derived from a probabilistic principal component analysis (pPCA). With TB‐MBLA marker and the principal component 1 (PC1) values, we reevaluated the original stage IIa dose–response and stages Ib/IIa pharmacokinetics—pharmacodynamics (PK‐PD) exposure‐response analyses, applying linear and non‐linear mixed models, respectively. For TB‐MBLA, we could not detect an exposure‐response effect in the PK‐PD analysis, in contrast with CFU and TTP. When combining biomarkers, we observed a significant but less pronounced *E*_max_ exposure–response between days 0–3 compared with CFU and TTP alone. We also successfully applied pPCA as a modeling framework and show evidence that combining CFU and TTP in a joint latent component can improve detection of treatment effects compared with either biomarker alone. In this study, we present novel EBA data for the first‐in‐class antimycobacterial compound BTZ‐043 and contextualize the value of emerging bacteriological markers within the EBA trial framework.

**Trial Registration:** ClinicalTrials.gov identifier: NCT04044001

## Study Highlights

- What is the current knowledge on the topic?
  ○ BTZ‐043 is a first‐in‐class antimycobacterial compound with promising applications in drug‐sensitive pulmonary tuberculosis. TB EBA trials are typically conducted using sputum culture assays for dose‐exposure‐response modeling, but the technical challenges of these assays limit their effectiveness.
- What question did this study address?
  ○ How does the novel RT‐qPCR assay TB‐MBLA perform as a dose‐exposure response marker for novel antitubercular compound BTZ‐043 and what are the effects of joint modeling approaches in EBA modeling tasks?
- What does this study add to our knowledge?
  ○ We did not detect a treatment effect for BTZ‐043 in TB‐MBLA over the 14‐day treatment window. Joint modeling of CFU and TTP markers with pPCA increases the statistical precision to detect a treatment effect compared with either marker alone and can overcome the issues of missing and contaminated data that are canonical of culture data from TB treatment monitoring cohorts.
- How might this change drug discovery, development, and/or therapeutics?
  ○ Designing EBA trials should consider the drug‐bacterial subpopulation axis of the specific study drug to maximize efficiency. Latent variable modeling techniques can be an effective and efficient framework for modeling *Mycobacterial tuberculosis* load in EBA trials.

## 1 Introduction

A pivotal step in tuberculosis (TB) drug development is the early bactericidal activity (EBA) study, designed to evaluate the anti‐mycobacterial effects of novel monotherapies or combination regimens over the first 7 or 14 days of treatment. At present, the biomarkers used to monitor treatment response are culture‐based, colony forming units (CFU) on solid media and time‐to‐positivity (TTP) in liquid media. The technical complexity, susceptibility to contamination, data variability [1] and long time to results [2] have motivated the search for alternative treatment monitoring methods. In this context, the Tuberculosis Molecular Bacterial Load Assay (TB‐MBLA), a real‐time quantitative polymerase chain reaction (RT‐qPCR)‐based method, has emerged as a promising non‐culture alternative for assessing treatment response. It has demonstrated applicability as a non‐culture‐based treatment monitoring assay [3] and could overcome many limitations of culture‐based biomarkers such as long incubation times and risk of contamination.

Each microbiological assay captures distinct but overlapping bacterial subpopulations of *Mycobacterium tuberculosis* within the host [1, 4, 5]. These subpopulations differ in treatment response and growth dynamics [6, 7]. CFU is a direct measure of the cultivable bacteria and TTP is an indirect time‐to‐event measure of dividing and aerobically metabolizing bacteria. In contrast, TB‐MBLA quantifies the target 16 s‐rRNA, representing a quantification of all viable bacteria [8, 9]; therefore, this assay could quantify additional non‐cultivable populations otherwise missed by solid CFU and liquid TTP culture methods. While these biomarkers are often modeled separately, joint analysis may reveal shared dynamics of the overall bacterial population or highlight common treatment‐response signals across assays [5].

We generated TB‐MBLA data from participants of the PanACEA‐BTZ‐043‐02 phase Ib/IIa trial dose‐ranging trial of BTZ‐043, one of several novel TB drug candidates currently in clinical development [10, 11]. The study enrolled 78 patients across 2 sites in South Africa, resulting in paired TB‐MBLA, CFU, and Mycobacterial Growth Indicator Tube (MGIT) TTP data for joint analysis. The primary results of the CFU and MGIT TTP data have been published elsewhere [12, 13]. We then conducted a proof‐of‐concept analysis by applying a simple machine learning‐based approach for joint modeling to the paired data that is robust to missing data. The aims of this study were two‐fold: to examine how TB‐MBLA performs as a marker of antimycobacterial drug efficacy; and to evaluate the effect of combining microbiological assay data on the precision of model estimates.

## 2 Methods

### 2.1 Patient Recruitment and Study Design

All participants provided written informed consent, and the trial was approved by all required ethics committees (PharmaEthics: reference 190622615; Medical Faculty of LMU Munich: reference 19‐0550; South African Health Products Regulatory Authority, reference 20190606).

The design and patient recruitment of the PanACEA‐BTZ‐043‐02 phase Ib/IIa trial are described in detail in the main study publication [12]. Briefly, 24 and 54 patients were recruited in Phase Ib and IIa, respectively. In phase Ib, patients were assigned to ascending dose groups of BTZ‐043 with daily doses of up to 1750 mg, with 3 patients per group and 6 patients in the highest dose group. In phase IIa, patients were randomized (3:3:3:2) to receive either 250, 500, or 1000 mg BTZ‐043 daily or standard of care (HRZE).

For the validation dataset, we leveraged publicly available MGIT TTP and solid agar CFU data from the NC‐003‐C‐J‐Pa‐Z trial, an EBA study examining the bactericidal activity of clofazimine (C) and pyrazinamide (Z) monotherapies, and 4 separate combination arms comprising bedaquiline (B) and/or pretomanid (Pa) (B‐Z‐C; B‐Pa‐Z; B‐Pa‐Z‐C; B‐Pa‐C) across 2 weeks. Full details on the sampling methods and microbiological assay protocols can be found in the original publication [14]. Data were accessed via the open‐source TB‐Platform for Aggregation of Clinical TB Studies repository [15].

### 2.2 Bacterial Load Determination

During the trial, overnight sputum samples were obtained at Screening and Days 0, 2, 3, 4, 6, 8, 11 and 14 of treatment. Samples were homogenized and aliquoted into separate vials, each used once for a separate assay. As part of the original trial [12], solid culture CFU counts were obtained from ten‐fold dilutions of digested sputum inoculated on Middlebrook 7H11 selective agar plates (Media Mage, Johannesburg, South Africa). TTP was assessed from decontaminated sputum samples using the BACTEC MGIT 960 system (Becton Dickinson, Johannesburg, South Africa). These assays were performed at the mycobacterial laboratory of TASK (South Africa). Assay data were log‐transformed to approximate a normal distribution for downstream modeling tasks. Limits of quantification was set at 1.0 CFU/mL for solid CFU, and 600 h (25 days) for MGIT TTP [16].

For the present study, frozen stored sputum samples with RNA stabilized by guanidinium isothiocyanate/ß‐mercaptoethanol were processed using the LifeArc TB‐MBLA PCR assay according to manufacturer guidelines [17] at the National Institute for Medical Research‐Mbeya Medical Research Center. PCR run‐specific standard curves were generated to convert PCR quantification cycle (Cq) values to Starting Quantity (SQ) values, which are the number of copies of TB 16S per reaction (c/rxn). The SQ values represent the bacterial load and were log‐transformed. The lower limit of detection was set at 128 copies per reaction (c/rxn).

### 2.3 Probabilistic Principal Component Analysis (pPCA)

To derive a representation of the joint distribution each biomarker assay, we used probabilistic principal component analysis (pPCA), a probabilistic variant of PCA formulated as a factor‐analysis model (Equation 1), in which the data are assumed to arise from a lower‐dimensional latent variable structure:

(1) $$t=\mathrm{Wx}+\mu +\epsilon ,\;\epsilon ∼N\left(0,\sigma^{2}I\right)$$

Here, **t** is the observed data, **x** the vector of latent variables, **W** the factor loading matrix, and **e** follows an isotropic Gaussian distribution, with **I** as the identity matrix. Traditional PCA seeks to reduce the dimensions of the original data into a subspace defined by the directions of highest variation using eigen decomposition of the sample covariance matrix. pPCA extends this by assuming a latent variable structure; here we estimate the conditional probability density of the observed data (Equation 2), and subsequently the latent variables (Equation 3).

(2) $$t|x∼N\left(\mathrm{Wx}+\mu ,\sigma^{2}I\right)$$

$$x|t∼N\left(M^{-1}W^{T}\left(t-\mu \right),\sigma^{2}M^{-1}\right),$$

(3) $$M=W^{T}W+\sigma^{2}I$$

We obtain the principal components (PCs) loadings from the columns of **W**, and the PC scores per patient from **x**, through calculation of the mean of the posterior distribution (Equation 3) via Bayes theorem. Importantly, the probabilistic framework is applicable to datasets with missing data points [18], allowing inclusion of all patients with at least one available assay measurement at a given time point. Full details on the model and parameter estimation can be found elsewhere [18]. We applied the pPCA framework to compute the first principal component (PC1) from paired CFU, TTP, and TB‐MBLA data, and then all combination pairs of CFU, TTP and TB‐MBLA, using the pca‐magic Python package [19].

### 2.4 Dose‐Exposure Response Modeling and Statistical Analysis

After computing the PC1 values for each patient, we compared these values with the three separate biomarkers in the dose‐exposure‐response models from the original analysis.

#### 2.4.1 Dose–Response

Descriptive dose response modeling was performed as in the original study [12], using linear mixed effect methods, with random intercepts and random slopes in R (version 4.3.3). To assess the precision of model estimates, we calculated the coefficient of variation (CV) (Equation 4):

(4) $$\mathrm{CV}=\frac{\mathrm{SE}\left(\hat{\beta }\right)}{\left|\hat{\beta }\right|}\times 100,$$

where is the model coefficient and SE represents its standard error.

For the validation dataset, we constructed additional linear mixed models with random intercepts and random slopes to assess the dose–response of the study arms. For these data, calculating the CV values for each model estimate became unreliable due to the presence of near‐zero coefficient values, leading to unstable CV estimates. Therefore, we compared model estimate precision via the standard errors of the model parameter estimates, following standardization of the raw data.

#### 2.4.2 Exposure‐Response

For the PK‐PD exposure‐response analysis, the relationships between BTZ‐043_total_ AUC_0‐24_ and the TB‐MBLA and PC1 values were modeled using non‐linear mixed effects methodology. BTZ‐043_total_ represents the sum of BTZ‐043 and its instable metabolite M2 [20], and the AUC values were derived from the population PK model developed as part of the original analysis [13]. In phase Ib, the AUC_0‐24_ values were determined on Day 12, and in Phase IIa on Day 14.

The full PK‐PD modeling process has been described in detail in the original study analysis [13]. Briefly, we examined linear and non‐linear [21] exposure‐response relationships for the TB‐MBLA biomarker and PC1 values and evaluated model fit by beginning with the simplest (linear) fit and testing successively complex exposure relationships via a likelihood ratio test (LRT) of the objective function value (OFV). Interindividual variability (IIV) was assumed to be log‐normally distributed for the TB‐MBLA data. The IIV for the intercept of PCA data was estimated on normal scale. The residual error model was additive on the log scale.

Stepwise covariate modeling was also performed as in the original analyses [13], to examine the effect of baseline covariates on the exposure‐response relationship. Age, HIV status, and site were included.

As a qualitative measurement of statistical precision, we compared the magnitude of the decrease in OFV (dOFV) for the model selection process between the single and combined biomarkers. All modeling was performed using NONMEM 7.5 with Pirana 2.9.9 as graphical interface, and Perl‐speaks‐NONMEM 5.3.0 for additional functionalities. Visual predictive checks were employed to evaluate model fit. Model selection was performed using −3.84 dOFV as the threshold for significance for the LRT for model fit (corresponding to an alpha level of 0.05 [*p* < 0.05]). Data management was performed using Python 3.10 in VSCode.

## 3 Results

### 3.1 Bacteriological Assays Throughout Treatment

Data from 78 participants across 8 timepoints were included. There were 0% contamination with the TB‐MBLA assay, compared with 0.9% in MGIT TTP and 8.4% in solid CFU. All cases of missingness in the TB‐MBLA were due to lack of sample availability. There were 2 TTP and 7 CFU samples above their respective limits of quantification, which were removed. There were no TB‐MBLA datapoints below the limit of quantification. Tables of the summary statistics of all assays over time in each treatment group are included in the Supporting Informations (Tables S1 and S2). Figure 1 shows the per‐patient trajectories for each bacteriological assay and the PC1 values in each phase.

**FIGURE 1:**
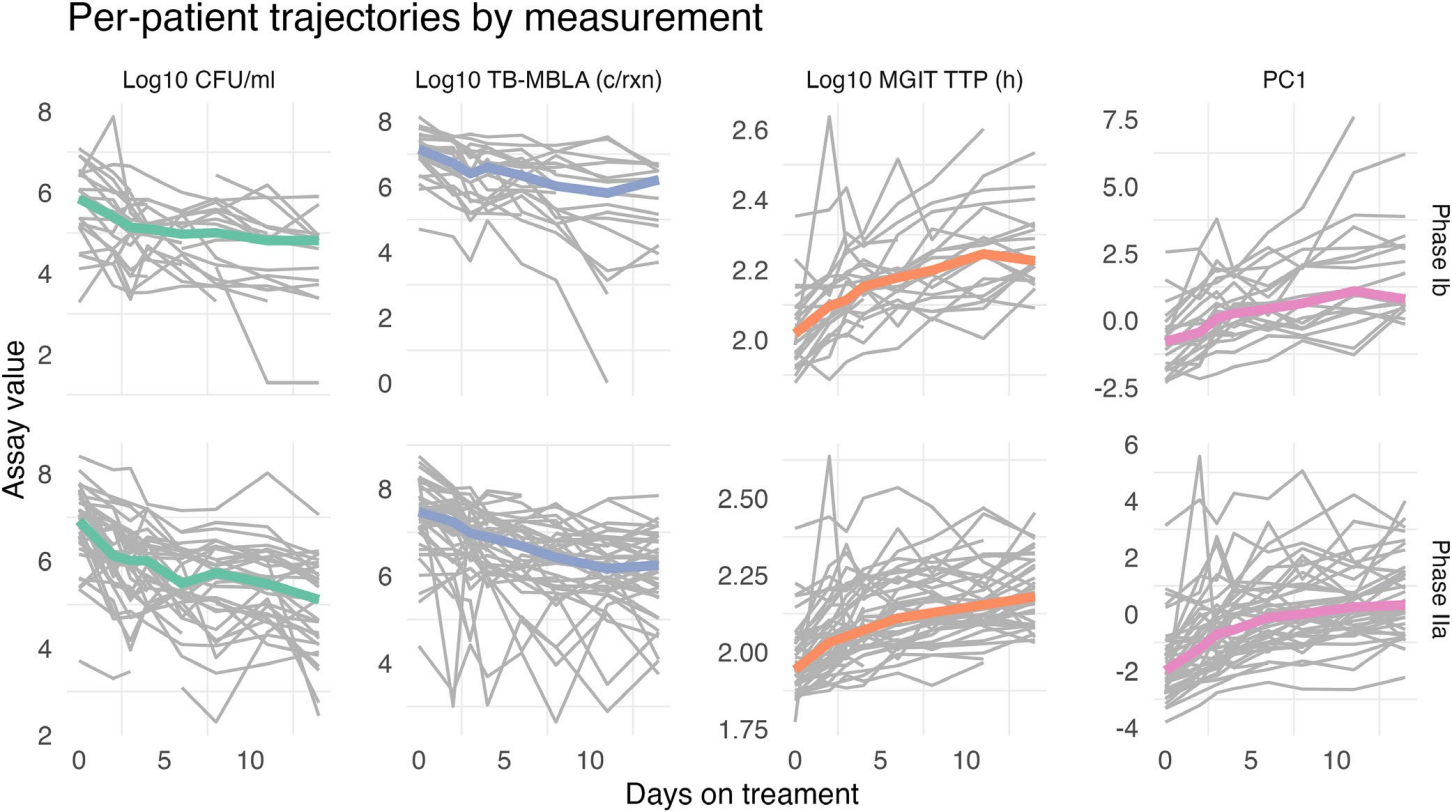
Spaghetti plots of per‐patient trajectories in each biomarker and PC1 values over time, by phase. The colored lines represent the median over time. Breaks in individual lines represent missing data, either from sample availability or bacterial contamination.

### 3.2 PC1 as a Measure of Bacterial Burden

In our data, the first PC (PC1) explained a high proportion (86%) of the total variance of the data and was informed equally by each individual assay, as indicated by the similar magnitudes of loadings. Additionally, the direction of the loadings reflect the patterns observed in the original assays; the positive loading for MGIT TTP indicates that high MGIT TTP values correspond with high PC1 scores, and higher CFU and TB‐MBLA assay values would result in lower PC1 values (Figure 2; Table 1). In context, lower PC1 values represent higher bacterial load, and higher PC1 values represent lower burden. As such, we hypothesized that PC1 represented a good summary metric of the joint distribution, with lower values indicating higher bacterial load. The second PC (PC2) represents the axis of second highest variance and is orthogonal to PC1; from the loadings, we observed that PC2 was primarily informed by TB‐MBLA but did not explain a high amount of variance. Therefore, we considered only PC1 values for our downstream modeling tasks.

**FIGURE 2:**
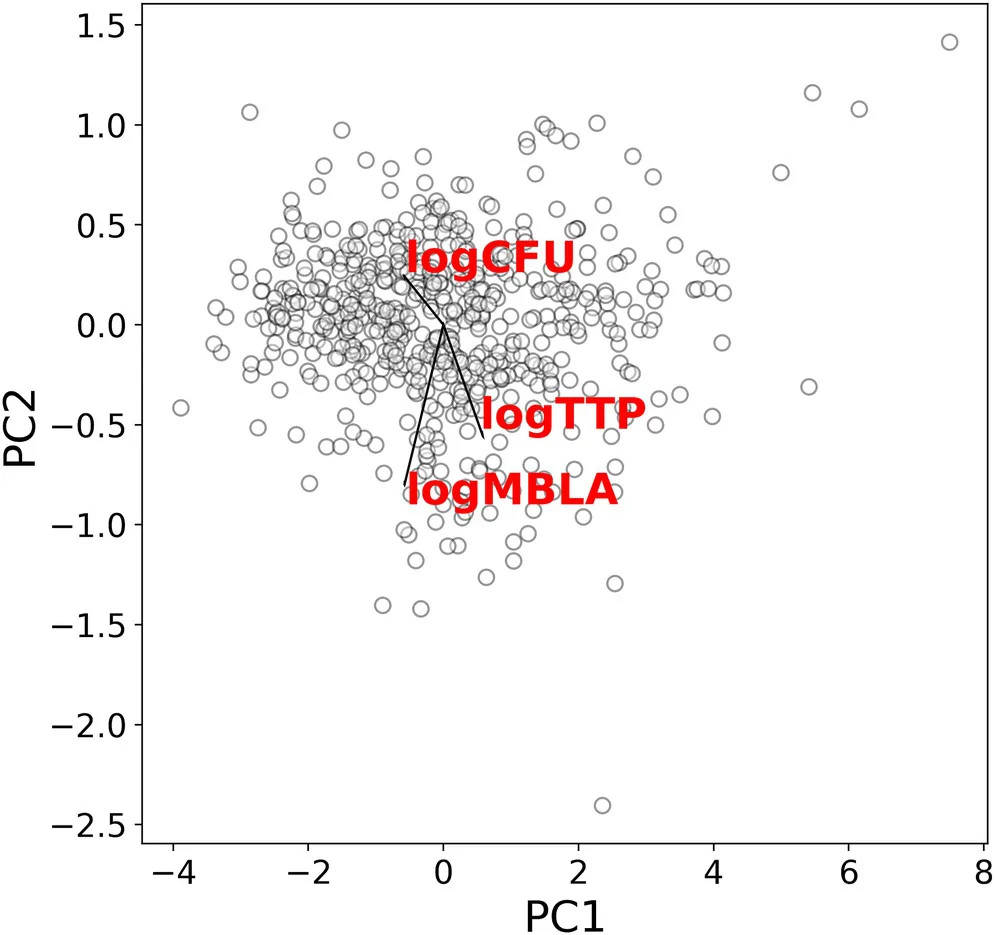
Probabilistic Principal Component Analysis biplot. Each datapoint is from an individual patient at a single timepoint. The black lines—vectors—represent the relationship between the original variables on the first 2 PCs. Along the PC1 axis, the direction of the TB‐MLBA and CFU vectors indicate that these biomarkers have negative loadings on this PC, and the MGIT TTP vector denotes a positive loading.

**TABLE 1:** Principal component loadings and proportion of variance explained by each PC.

PC	Loadings			Total variance explained
	CFU	MGIT TTP	TB‐MBLA	
PC1	−0.545	0.602	−0.584	0.860
PC2	0.595	−0.208	−0.774	0.079

*Note:* The loadings—which are equivalent to correlation coefficients—range from −1 to 1. Their magnitude represents how much a single variable contributes to the PC, and the sign indicates the direction of the association. The total variance explained indicates how well each of the principal components captures the overall variance within the data (between 0 and 1). A higher value indicates a higher proportion of variance is explained by the individual PC.

Abbreviations: CFU, colony forming units; MGIT TTP, Mycobacterial Growth Indicator Tube Time to Positivity; TB‐MBLA, Tuberculosis Mycobacterial Load Assay.

### 3.3 Dose Response Models

We reproduced the dose–response modeling performed in the original study using the TB‐MBLA and PC1 markers. Figure 3 reports the coefficients from the linear mixed model fixed effects, which represent the bactericidal slopes of each arm.

**FIGURE 3:**
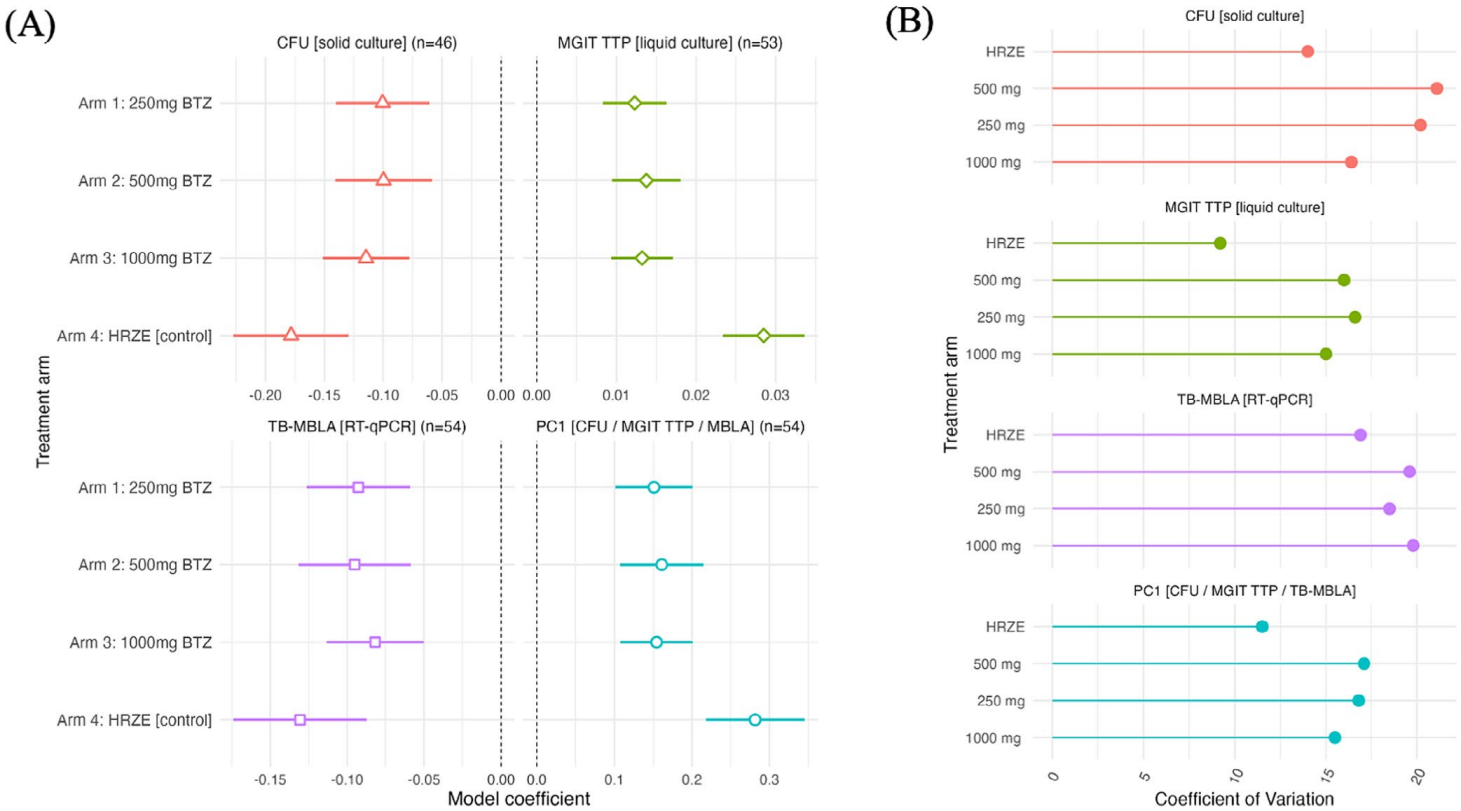
Precision of linear mixed effect modeling coefficients for individual assays and PC1 values. Linear mixed models were constructed with a treatment‐by‐time interaction term as a fixed effect. (A) The resulting coefficients from the fixed effect represent the average slope per treatment arm. For MGIT TTP and PC values, a positive slope represents bactericidal activity, with greater values representing greater effect. Conversely, for CFU and TB‐MBLA markers, a negative slope indicates presence of bactericidal activity, with greater values indicating greater effect. (B) CV values for each model coefficient. A smaller CV indicates greater precision of the model estimate. CFU, colony forming units; CV, coefficient of variation; MGIT TTP, Mycobacterial Growth Indicator Tube Time to Positivity; TB‐MBLA, Tuberculosis Mycobacterial Load Assay.

As a measure of estimate precision, the CV for each treatment arm slope from each assay was calculated (Figure 3B; Table S3). TB‐MBLA was comparable to solid CFU in terms of estimate precision, and MGIT TTP exhibited the greatest precision. The PC1 biomarker was more precise than the CFU and TB‐MBLA single‐assay models across all arms. The forest plots and CV values of the linear mixed models for all iterations of the pPCA algorithm (CFU + MGIT TPP; CFU + TB‐MBLA, TB‐MBLA + TTP) can be found in Figures S1 and S2.

### 3.4 Exposure‐Response Identified in PC1 Marker, but Not TB‐MBLA

The TB‐MBLA and PC1 markers were then examined in a PK‐PD exposure‐response framework. The complete model parameters for all models built are included in Tables S4–S6.

#### 3.4.1 TB‐MBLA

The change in TB‐MBLA log10 c/rxn over time was best described using a bi‐linear (stepwise linear) mixed model (LRT *p* < 0.001) with a random intercept, suggesting a change in the rate of bacterial decline over time. We estimated the node at 2 days, similar to what was found in the CFU and MGIT TTP models from the previous analysis [11]. Figure 4A shows a visual predictive check (VPC) indicating that the model fit the data well. We identified no significant exposure response of the BTZ‐043_total_ AUC_0‐24_ on either of the slopes, or over the entire 14‐day treatment duration. We also assessed the relationship between TB‐MBLA log10 c/rxn and BTZ‐043 (parent); again, no exposure‐response relationship could be identified. No significant association was observed between the baseline covariates on the model parameters.

**FIGURE 4:**
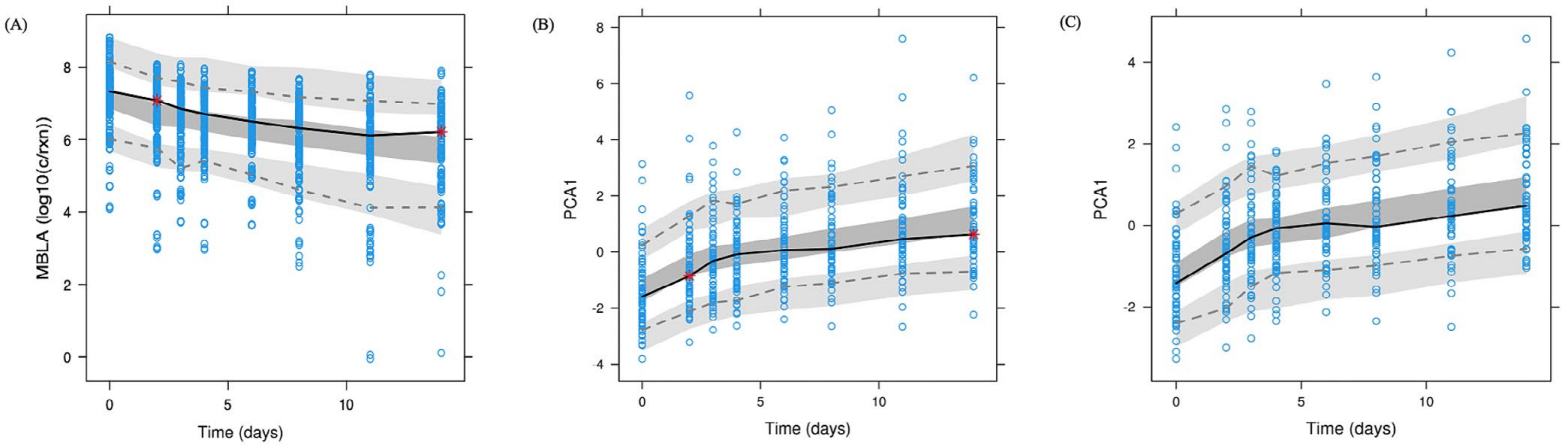
Visual predictive check plots of (A) TB‐MBLA only, (B) 3‐marker PCA (TTP + CFU + TB‐MBLA) and (C) 2‐marker PCA (TTP + CFU). Plots examining the model fit of each exposure‐response model via simulations. Unbroken black line represents the median values per timepoint from the observed data, and the dashed lines the 5th and 95th percentiles of the observed data per timepoint. Gray areas represent the 95% confidence intervals for the 5th and 95th percentiles, and the median, of the simulated data. Red points indicate where the observed median of the data falls outside of the range of predicted values.

#### 3.4.2 pPCA Metric

We also applied the same model selection method to the PC1 values for each patient; a bi‐linear model with the node at 3.2 days, with a significant exposure‐response of BTZ‐043_total_ AUC_0‐24_ on the first slope, was selected as the best‐fitting model (Figure 4B), mirroring what was observed in the prior PK‐PD analysis.

#### 3.4.3 Exploring Other Biomarker Combinations

Given the results from the TB‐MBLA PK‐PD model, we considered if the results observed in the PC1 model derived from all 3 assays may have been driven by signal in the CFU and MGIT TTP data. As such, we repeated the pPCA analysis using only CFU and MGIT TTP values and modeled the resulting PC1 as before. A bi‐linear model with the node estimated at 2.8 days was selected as the best model, and significant exposure response with the BTZ‐043_total_ AUC_0‐24_ was identified on the first slope, until day 3 (Figure 4C). Table 2 reports the results of our qualitative comparison of precision. A greater decrease in test statistic—here the objective function value (OFV) – indicates a greater effect size, translating to increased precision. The full table with all pPCA iterations is listed in Table S7.

**TABLE 2:** Summary of exposure‐response effects for BTZ‐043 using different output markers.

Bacteriological markers			Effect	ΔOFV (*p*‐value)
CFU	TTP	MBLA		
X			BTZ‐043_total_ AUC_0‐24_ *E*_max_	−11.30 (*p* = 3.5 × 10^−3^)
	X		BTZ‐043_total_ AUC_0‐24_ *E*_max_	−11.11 (*p* = 3.9 × 10^−3^)
		X	BTZ‐043_total_ AUC_0‐24_ linear	0 (NA)
X	X		BTZ‐043_total_ AUC_0‐24_ *E*_max_	−12.55 (*p* = 3.9 × 10^−4^)
X	X	X	BTZ‐043_total_ AUC_0‐24_ *E*_max_	−5.535 (*p* = 0.0186)

*Note:* The table presents the ΔOFV and corresponding *p*‐values for exposure‐response relationships analyzed with MGIT TTP, CFU, TB‐MBLA and all pairwise combinations derived via pPCA. The combined MGIT TTP‐CFU marker demonstrates the greatest statistical resolution, emphasizing its potential to enhance analysis in early bactericidal activity (EBA) trials. *E*_max_ refers to the classic *E*_max_ parameterization of the exposure‐response model.

Abbreviations: ΔOFV, change in objective function value; CFU, colony forming units; MGIT TTP, Mycobacterial Growth Indicator Tube Time to Positivity; pPCA, probabilistic PCA; TB‐MBLA, Tuberculosis Mycobacterial load assay.

When comparing all individual and combinations of biomarkers, the combination with the greatest ΔOFV was the CFU + MGIT TTP (−12.55), greater than both MGIT TTP and CFU alone. We also performed pPCA on all other possible combinations of biomarkers (CFU + TB‐MBLA; MGIT + TB‐MBLA). Addition of TB‐MBLA resulted in a significantly smaller ΔOFV in all iterations, and there was no evidence to suggest an exposure‐response in either the MGIT + MBLA or CFU + MBLA‐derived PC1 markers, following the same model selection process outlined above.

#### 3.4.4 pPCA Framework Generalizes Across Trials and Antimycobacterial Compounds

In the validation dataset, there were 53 and 16 negative CFU and MGIT TTP cultures, respectively, which were removed. Of the remaining data, 3.7% CFU and 3.0% MGIT TTP datapoints were missing due to technical reasons (contamination or missing samples). The per‐patient trajectories of raw CFU, MGIT TTP, and PC1 data (Figure S3) show that the PC1 values change over time in the same direction as MGIT TTP, similar to the original dataset. Encouragingly, the PC loadings for this iteration also showed that the 1st PC was equally informed by both assays, as seen in the first dataset, and explained 84% of the variance of the data (Table 3). We observed a signal of treatment effect in the PC1 values via the non‐zero slopes from dose–response model estimates (Figure 5); while the PC1 estimates were more precise than CFU, they were not as precise as MGIT TTP (Table 4), mirroring the results observed in the dose–response models from our original dataset.

**TABLE 3:** Principal component loadings and proportion of variance explained by each PC in the validation dataset.

PC	Loadings		Total variance explained
	CFU	MGIT TTP	
PC1	−0.694	0.716	0.84

*Note:* The loadings—which are equivalent to correlation coefficients—range from −1 to 1. Their magnitude represents how much a single variable contributes to the PC, and the sign indicates the direction of the association. The total variance explained indicates how well each of the principal components captures the overall variance within the data (between 0 and 1). A higher value indicates a higher proportion of variance is explained by the individual PC.

Abbreviations: CFU, colony forming units; MGIT TTP, Mycobacterial Growth Indicator Tube Time to Positivity; TB‐MBLA, Tuberculosis Mycobacterial Load Assay.

**FIGURE 5:**
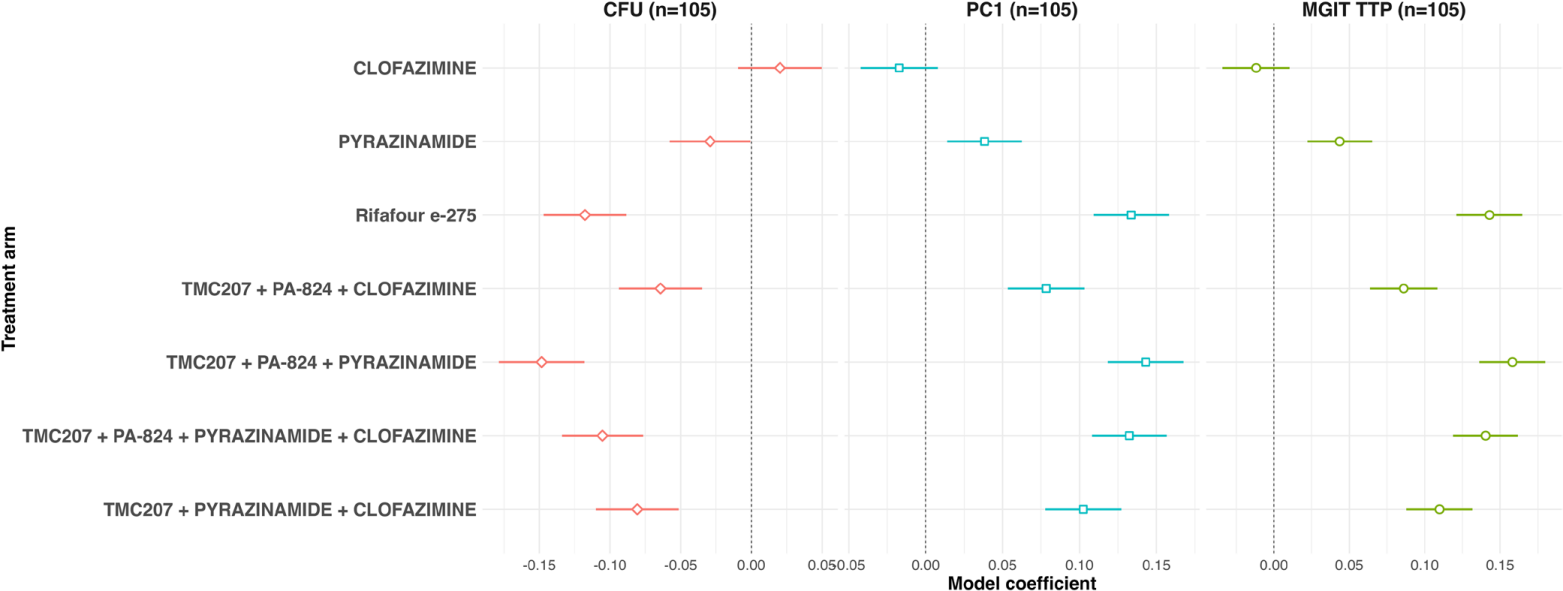
Precision of linear mixed effect modeling by assay in the validation dataset. Linear mixed models were constructed with a treatment‐by‐time interaction term as a fixed effect. The resulting coefficients from the fixed effect represent the average slope per treatment arm. For MGIT TTP and PC1 datapoints, a positive slope represents bactericidal activity, with greater values representing greater effect. MGIT TTP, Mycobacterial Growth Indicator Tube Time to Positivity; PC1, 1st principal component.

**TABLE 4:** Standard errors of per‐arm slope estimates for each measurement in the validation dataset.

Treatment arm	CFU	MGIT TTP	PC1
Clofazimine	0.0149	0.0114	0.0126
Pyrazinamide	0.0143	0.0109	0.0122
Rifafour e‐275	0.0148	0.0111	0.0123
TMC207 + PA‐824 + Clofazimine	0.0148	0.0114	0.0126
TMC207 + PA‐824 + Pyrazinamide	0.0153	0.0111	0.0123
TMC207 + PA‐824 + Pyrazinamide + Clofazimine	0.0144	0.0110	0.0122
TMC207 + Pyrazinamide + Clofazimine	0.0148	0.0112	0.0125

*Note:* The columns list the magnitude of the standard errors for each slope estimate for each treatment group. A smaller standard error indicates greater precision. Across all treatment groups, the estimates from the MGIT TTP model are the most precise, followed by the PC1 model.

Abbreviations: CFU, colony forming units; MGIT TTP, Mycobacterial Growth Indicator Tube Time to Positivity; PA‐824, pretomanid; TMC207, bedaquiline.

## 4 Discussion

In the present study, we found that there was no observable exposure‐response signal in TB‐MBLA for the antimycobacterial compound BTZ‐043 over the first 2 weeks of treatment, in contrast with solid CFU and liquid MGIT TTP. We also demonstrated the utility of pPCA as a simple machine learning framework for modeling joint distributions that increased statistical resolution in PK‐PD models compared with single markers, and that this approach was generalizable to across trial datasets and different antimycobacterial compounds.

In the PK‐PD modeling in the present study, we did not observe an exposure response in the TB‐MBLA data, which contrasted with the results from the previous PK‐PD analysis in CFU and MGIT TTP alone. Additionally, when the TB‐MBLA data were combined with CFU and MGIT TTP in the pPCA framework, the model estimates became less precise than with CFU or MGIT TTP alone. Contextualizing these results requires consideration of the bacterial subpopulation dynamics during infection and treatment: Heterogeneity in bacterial subpopulations is observed within [22] and between [23] individuals with active TB infection. Concomitantly, separate subpopulations may be differentially identifiable depending on the assay selected, highlighted by discordance observed between culture‐based methods (CFU and MGIT TTP) and TB‐MBLA in both clinical trial data [24] and recent murine models [25]. Taken together, this suggests that whilst these assays capture largely overlapping populations, the specific types of bacterial subpopulations detectable by culture and non‐culture‐based assays may diverge in important ways throughout the treatment period; in the context of the present study, if participants initiated the trial with low numbers of persistent bacteria in the sputum, it would follow that the signal within the TB‐MBLA data would be limited until the visibility of any persister populations increased.

Furthermore, it is also important to consider recent data showing the lesion penetration capabilities and antimycobacterial effects of BTZ‐043 within the necrotic centre of murine lesions [26]. Here, the authors observed appreciable decreases in bacterial load within the lesion as measured by TB‐MBLA, but with very little difference observed after 2 weeks and the greatest difference observed after 4–6 weeks of therapy. In the context of the present study, it could be that the 2‐week timeframe of the standard EBA study is not sufficient to fully capture the bactericidal activity of BTZ‐043 as measured by TB‐MBLA. Finally, the lack of observable signal could also be due to increased noise within the assay, obscuring any treatment effect. Future investigation of TB‐MBLA data from longer studies with different compounds will help to determine the capacity of TB‐MBLA for measurement of antimycobacterial drug activity over longer periods.

Modeling approaches incorporating multiple biomarkers have been posited to simultaneously describe differential treatment effects in the different bacterial subpopulations present [5, 27, 28]. The PC1 from the pPCA model in the present study represents the direction of greatest variance of the dataset and was a good summary metric of the overall distribution. We hypothesized that changes in PC1 may mirror a general treatment effect of a given compound that is informed by each individual marker, which was supported by our observation of a positive exposure‐response signal in the PC1 data in the original dataset, and the successful recovery of the EBA signal within a dose–response modeling framework in independent data. The observed increase in resolution of the exposure‐response signal in the PC1 data compared with the single biomarkers suggests that consolidation of shared signal via this latent variable framework is an efficient method of describing treatment effect within bacterial subpopulations. Interestingly, contrasting the results of the dose–response and exposure‐response models may also offer insights into optimal endpoints for each task; the increase in resolution of the PC1‐based exposure‐response endpoint compared with the separate assays suggests that the overall shared variance between culture assays provides a cleaner estimate of the underlying patient‐level exposure‐response relationship, but may not be optimized for estimating group‐wise dose response parameters.

The present study has several limitations: pPCA relies on gaussian distributional and temporal linearity assumptions, which may not reflect the true temporal covariance of the biomarkers [29, 30]. The impact may be limited in this case given the short timeframe of the study, but this might not hold in longer studies. These assumptions carry over to the PK‐PD modeling task in which we assume the same exposure‐response in each of the biomarkers. This is supported by evidence from the original description of the PK‐PD response in each individual assay and has been observed for other drugs like rifampicin [13]. However, we concede that this assumption may prove inadequate when considering this method for other anti‐mycobacterial compounds. Finally, we note that the architecture of the dose–response model built for the validation data approximates the original Bayesian hierarchical non‐linear mixed (NLME) model described by the authors; however, these authors also note that their results obtained via the NLME method were “very similar” when tested with more simple regression methods [14], suggesting that the model parameters obtained in our analysis remain a valid estimate of the true treatment effect in these data.

Despite the technical advantages of TB‐MBLA over traditional culture methods, we did not observe an exposure‐response signal for BTZ‐043_total_ or BTZ‐043, and these data did not add to the explanatory power of the combined biomarker models. However, we demonstrate that joint modeling via pPCA represents a practical approach to improve resolution of dose‐exposure‐response signal in culture‐based biomarkers within an EBA study. Our pPCA‐based approach increased the resolution of the exposure‐response signal within an EBA study setting and proved robust to data missing at random, which is a common and limiting reality of culture‐based treatment monitoring. Further work is required to develop this joint modeling framework to include more permissive inter‐biomarker relationships, including non‐linear relationships, and to examine the potential applications of this method in longer Phase II trials.

## Author Contributions

M.C.W.M., S.E.K., J.D., S.M.‐M., P.P.J.P, V.R.D.J., R.D., K.N., A.H.D., N.H., E.M.S., and F.P.C., wrote the manuscript. M.H. and F.P.C. designed the research. M.C.W.M. and S.E.K. performed the research and analyzed the data. F.N., D.M., B.M., and E.M.S. contributed new reagents/analytical tools.

## Funding

N.H., V.R.D.J., J.D., R.D., K.N., E.M.S., P.P.J.P., A.H.D., and M.H. received grants from the European and Developing Countries Clinical Trials Partnership (EDCTP), the German Ministry of Education and Research (BMBF), the German Center for Infection Research (DZIF), and the Bavarian Ministry for Science and the Arts to their institutions. F.P.C. was funded by the Free State of Bavaria's Hightech Agenda through the Institute of AI for Health (AIH). This project has received funding from the Innovative Medicines Initiative 2 Joint Undertaking (JU) under grant agreement No. 101007873. The JU receives support from the European Union's Horizon 2020 research and innovation programme and EFPIA, Deutsches Zentrum für Infektionsforschung e. V. (DZIF), and Ludwig‐Maximilians‐Universität München (LMU). EFPIA/AP contribute to 50% of funding, whereas the contribution of DZIF and the LMU University Hospital Munich has been granted by the German Federal Ministry of Education and Research. The original study was part of the EDCTP2 programme (grant TRIA2015‐1102) and funded by the German Ministry for Education and Research (01KA1701); German Center for Infection Research; InfectControl (03ZZ0803A, 03ZZ0835A, and 03ZZ0826A0); Bavarian Ministry for Science and the Arts; Swiss State Secretariat for Education, Research, and Innovation; and Nederlandse Organisatie voor Wetenschappelijk Onderzoek.

## Conflicts of Interest

N.H., J.D., E.M.S., P.P.J.P., and M.H. received funding from LifaChem Biosciences for the evaluation of delapazolid to their institutions. E.M.S. received funding to the institution from Janssen Pharmaceuticals for a scientific project related to bedaquiline and from TB Alliance for projects related to pediatric development of pretomanid.

## Supporting information


**Figure S1:** Forest plot for fixed effect coefficients from linear mixed effect modeling for all iterations of PCA.
**Figure S2:** Coefficient of variation values for fixed effect coefficients from linear mixed effect modeling for all iterations of PCA.
**Figure S3:** Spaghetti plots of per‐patient trajectories in CFU, MGIT TTP and PC1 over time in the validation dataset.
**Table S1:** Summary statistics by assay, by treatment arm for Phase Ib.
**Table S2:** Summary statistics by assay, by treatment arm for Phase IIa.
**Table S3:** CVs for the fixed effects from the dose–response mixed effect models.
**Table S4:** PK‐PD model parameters for TB‐MBLA.
**Table S5:** PK‐PD model parameters for PC1 (MGIT TTP + CFU + TB‐MBLA).
**Table S6:** PK‐PD model parameters for PCA1 (MGIT TTP + CFU).
**Table S7:** Summary of exposure‐response effects for BTZ‐043 using different output markers.

## Data Availability

The code to reproduce the pPCA model can be found in the study GitHub repository (https://github.com/mairi‐mcclean/BTZ‐043_pPCA.git). Requests for access to the data generated as part of this study should be submitted to the corresponding authors.
