# Supplementary material for: Modeling Treatment Response in Tuberculosis Early Bactericidal Activity Trials
Source: CPT Pharmacometrics Syst Pharmacol. 2026 Aug 7;15(8):e70311. doi: 10.1002/psp4.70311 (PMC13733376; doi:10.1002/psp4.70311)
Supplement: Supplementary file 1 [file PSP4-15-e70311-s001.docx]

**Supplementary materials**

**Table S1. Summary statistics by assay, by treatment arm for Phase Ib**.

| **Assay** | **Day 0**  N=3*^1^* | **Day 2**  N=3*^1^* | **Day 3**  N=3*^1^* | **Day 4**  N=3*^1^* | **Day 6**  N=3*^1^* | **Day 8**  N=3*^1^* | **Day 11**  N=3*^1^* | **Day 14**  N=3*^1^* |
| --- | --- | --- | --- | --- | --- | --- | --- | --- |
| **Cohort 1: BTZ 250mg** | | | | | | | | |
| logCFU | 4.11 (3.28, 5.88) | 4.53 (4.25, 4.92) | 3.53 (3.35, 4.69) | 4.69 (3.53, 5.16) | 4.04 (3.68, 5.44) | 3.68 (3.31, 5.76) | 4.24 (3.69, 4.80) | 4.16 (3.40, 4.92) |
| Missing, n | 0 | 0 | 0 | 0 | 0 | 0 | 1 | 1 |
| logTTP | 2.16 (2.15, 2.35) | 2.15 (2.15, 2.37) | 2.19 (2.16, 2.43) | 2.18 (2.17, 2.28) | 2.21 (2.14, 2.39) | 2.25 (2.15, 2.45) | 2.27 (2.24, 2.60) | 2.24 (2.17, 2.31) |
| Missing, n | 0 | 0 | 0 | 0 | 0 | 0 | 0 | 1 |
| logMBLA | 5.92 (4.71, 7.13) | 5.25 (4.47, 6.03) | 5.60 (3.73, 6.48) | 5.47 (4.96, 6.67) | 5.88 (3.64, 6.29) | 5.32 (3.15, 6.95) | 4.72 (0.01, 6.17) | 5.10 (3.95, 6.25) |
| Missing, n | 0 | 1 | 0 | 0 | 0 | 0 | 0 | 1 |
| **Cohort 2: BTZ 500mg** | | | | | | | | |
| logCFU | 6.08 (6.04, 6.47) | 6.05 (5.48, 6.69) | 5.83 (5.67, 6.65) | 5.84 (5.10, 6.42) | 5.50 (4.97, 6.00) | 5.51 (5.01, 5.80) | 5.89 (4.93, 6.19) | 4.82 (4.81, 4.94) |
| Missing, n | 0 | 0 | 0 | 0 | 0 | 0 | 0 | 0 |
| logTTP | 1.97 (1.93, 1.99) | 2.00 (1.89, 2.06) | 2.02 (1.94, 2.10) | 1.99 (1.96, 2.15) | 2.04 (1.98, 2.09) | 2.08 (2.06, 2.17) | 2.09 (2.00, 2.25) | 2.16 (2.15, 2.23) |
| Missing, n | 0 | 0 | 0 | 0 | 0 | 0 | 0 | 0 |
| logMBLA | 7.56 (7.30, 7.86) | 7.53 (7.09, 7.55) | 7.47 (7.03, 7.61) | 7.32 (6.60, 7.53) | 7.26 (6.87, 7.58) | 7.07 (6.02, 7.26) | 7.46 (5.84, 7.53) | 6.47 (6.27, 6.61) |
| Missing, n | 0 | 0 | 0 | 0 | 0 | 0 | 0 | 0 |
| **Cohort 3: BTZ 750mg** | | | | | | | | |
| logCFU | 5.38 (5.19, 5.81) | 4.59 (4.51, 5.53) | 4.08 (3.93, 5.29) | 4.61 (4.39, 5.64) | 4.29 (3.80, 5.13) | 3.85 (3.68, 5.54) | 3.59 (3.32, 4.74) | 4.31 (3.72, 4.89) |
| Missing, n | 0 | 0 | 0 | 0 | 0 | 0 | 0 | 1 |
| logTTP | 2.07 (2.03, 2.09) | 2.16 (2.10, 2.19) | 2.18 (2.08, 2.31) | 2.13 (2.08, 2.27) | 2.30 (2.11, 2.34) | 2.37 (2.06, 2.39) | 2.39 (2.17, 2.43) | 2.40 (2.17, 2.44) |
| Missing, n | 0 | 0 | 0 | 0 | 0 | 0 | 0 | 0 |
| logMBLA | 6.97 (6.30, 7.01) | 6.39 (5.94, 6.50) | 6.14 (4.96, 6.34) | 6.36 (5.56, 6.84) | 5.24 (4.97, 5.99) | 4.86 (4.60, 6.02) | 5.22 (3.43, 5.81) | 4.97 (3.70, 5.65) |
| Missing, n | 0 | 0 | 0 | 0 | 0 | 0 | 0 | 0 |
| **Cohort 4: BTZ 1000mg** | | | | | | | | |
| logCFU | 5.36 (4.46, 6.91) | 5.33 (4.22, 6.32) | 4.99 (4.99, 5.69) | 4.95 (4.40, 5.70) | 5.11 (4.75, 5.48) | 4.38 (4.38, 4.38) | 3.88 (3.88, 3.88) | 3.38 (3.38, 3.38) |
| Missing, n | 0 | 0 | 0 | 0 | 0 | 0 | 0 | 0 |
| logTTP | 2.02 (1.92, 2.09) | 2.05 (1.95, 2.18) | 2.08 (2.08, 2.18) | 2.17 (2.12, 2.23) | 2.18 (2.18, 2.18) | 2.32 (2.32, 2.32) | 2.28 (2.28, 2.28) | 2.33 (2.33, 2.33) |
| Missing, n | 0 | 0 | 0 | 0 | 0 | 0 | 0 | 0 |
| logMBLA | 7.46 (6.87, 7.79) | 7.41 (5.75, 7.47) | 6.45 (5.83, 7.12) | 6.42 (5.63, 7.33) | 6.45 (6.40, 6.50) | 5.06 (5.06, 5.06) | 5.04 (5.04, 5.04) | 4.80 (4.80, 4.80) |
| Missing, n | 0 | 0 | 0 | 0 | 0 | 0 | 0 | 0 |
| **Cohort 5: BTZ 1250mg** | | | | | | | | |
| logCFU | 6.92 (6.45, 7.09) | 6.06 (5.99, 6.50) | 5.99 (5.66, 6.04) | 5.51 (5.20, 5.82) | 5.39 (5.23, 5.54) | 5.85 (5.34, 6.43) | 5.14 (5.00, 5.87) | 5.38 (4.85, 5.90) |
| Missing, n | 0 | 0 | 0 | 1 | 1 | 0 | 0 | 1 |
| logTTP | 1.96 (1.92, 2.23) | 2.06 (2.01, 2.13) | 2.10 (2.01, 2.27) | 2.15 (2.07, 2.21) | 2.14 (2.09, 2.14) | 2.13 (2.12, 2.20) | 2.16 (2.13, 2.29) | 2.22 (2.21, 2.23) |
| Missing, n | 0 | 0 | 0 | 0 | 0 | 0 | 0 | 0 |
| logMBLA | 7.61 (7.18, 7.81) | 7.43 (7.20, 7.46) | 6.89 (6.33, 7.10) | 7.06 (6.74, 7.07) | 6.71 (6.60, 7.14) | 6.60 (6.37, 6.72) | 6.27 (6.15, 6.75) | 6.49 (6.24, 6.71) |
| Missing, n | 0 | 0 | 0 | 0 | 0 | 0 | 0 | 0 |
| **Cohort 6: BTZ 1500mg** | | | | | | | | |
| logCFU | 5.15 (4.86, 6.39) | 5.78 (3.70, 7.87) | 3.95 (3.54, 5.50) | 3.93 (3.85, 5.16) | 4.86 (4.47, 5.25) | 4.16 (3.73, 5.21) | 3.95 (1.30, 4.83) | 3.82 (1.30, 4.61) |
| Missing, n | 0 | 1 | 0 | 0 | 1 | 0 | 0 | 0 |
| logTTP | 1.99 (1.94, 2.06) | 2.14 (2.06, 2.64) | 2.19 (2.11, 2.26) | 2.22 (2.17, 2.31) | 2.20 (2.18, 2.52) | 2.25 (2.23, 2.29) | 2.28 (2.24, 2.47) | 2.29 (2.26, 2.53) |
| Missing, n | 0 | 0 | 0 | 0 | 0 | 0 | 0 | 0 |
| logMBLA | 7.16 (6.34, 7.21) | 6.71 (5.88, 7.00) | 5.54 (5.19, 6.57) | 5.64 (5.45, 5.90) | 5.89 (5.87, 6.34) | 5.31 (5.28, 5.81) | 4.09 (2.72, 5.46) | 5.09 (5.02, 5.16) |
| Missing, n | 0 | 0 | 0 | 0 | 0 | 0 | 1 | 1 |
| **Cohort 7: BTZ 1750mg** | | | | | | | | |
| logCFU | 5.56 (4.59, 6.07) | 5.28 (5.02, 5.65) | 5.14 (5.11, 5.34) | 5.10 (4.65, 5.24) | 4.56 (4.37, 5.02) | 4.82 (4.21, 5.59) | 4.83 (4.08, 5.16) | 4.97 (4.14, 5.70) |
| Missing, n | 0 | 0 | 0 | 1 | 0 | 0 | 0 | 0 |
| logTTP | 1.98 (1.90, 2.04) | 2.06 (2.05, 2.11) | 2.08 (2.08, 2.17) | 2.13 (2.06, 2.14) | 2.17 (2.13, 2.24) | 2.19 (2.08, 2.25) | 2.11 (2.11, 2.38) | 2.21 (2.09, 2.32) |
| Missing, n | 0 | 0 | 0 | 0 | 0 | 0 | 0 | 0 |
| logMBLA | 7.02 (6.91, 7.41) | 6.67 (6.43, 6.73) | 6.40 (6.13, 7.03) | 6.71 (5.22, 7.18) | 6.21 (5.43, 6.72) | 6.45 (4.86, 7.09) | 4.99 (3.14, 6.84) | 6.22 (4.21, 6.68) |
| Missing, n | 0 | 0 | 0 | 0 | 0 | 0 | 1 | 0 |
| *^1^*Median (Q1, Q3). CFU, Colony Forming Units; MGIT TTP, Mycobacterial Growth Indicator Tube Time to Positivity; TB-MBLA, Tuberculosis Mycobacterial Load Assay; c/rxn, copies per reaction. | | | | | | | | |

|  | | Arm 1: BTZ 250mg | | | | | | | | | Arm 2: BTZ 500mg | | | | | | | | Arm 3: BTZ 1000mg | | | | | | | | | | Arm 4: HZRE | | | | | | | | |
| --- | --- | --- | --- | --- | --- | --- | --- | --- | --- | --- | --- | --- | --- | --- | --- | --- | --- | --- | --- | --- | --- | --- | --- | --- | --- | --- | --- | --- | --- | --- | --- | --- | --- | --- | --- | --- | --- |
| **Assay** | **Day 0** N=15^1^ | | **Day 2** N=14^1^ | **Day 3** N=14^1^ | **Day 4** N=15^1^ | **Day 6** N=13^1^ | **Day 8** N=13^1^ | **Day 11** N=13^1^ | **Day 14** N=13^1^ | **Day 0** N=14^1^ | | **Day 2** N=12^1^ | **Day 3** N=12^1^ | **Day 4** N=12^1^ | **Day 6** N=10^1^ | **Day 8** N=11^1^ | **Day 11** N=11^1^ | **Day 14** N=11^1^ | | **Day 0** N=16^1^ | **Day 2** N=16^1^ | **Day 3** N=16^1^ | **Day 4** N=16^1^ | **Day 6** N=15^1^ | **Day 8** N=15^1^ | **Day 11** N=15^1^ | **Day 14** N=14^1^ | **Day 0** N=9^1^ | | **Day 2** N=9^1^ | **Day 3** N=9^1^ | **Day 4** N=9^1^ | **Day 6** N=7^1^ | **Day 8** N=8^1^ | **Day 11** N=8^1^ | **Day 14** N=7^1^ | |
| logCFU | 6.57 (6.15, 7.17) | | 6.38 (4.76, 6.90) | 6.39 (4.85, 6.60) | 6.10 (5.44, 6.30) | 5.55 (4.69, 6.18) | 5.78 (4.12, 6.34) | 5.60 (4.64, 6.04) | 5.57 (4.10, 5.88) | 7.05 (6.43, 7.34) | | 6.19 (5.50, 6.86) | 5.89 (5.39, 6.20) | 5.91 (5.38, 6.73) | 5.33 (5.21, 5.60) | 5.57 (4.88, 6.28) | 5.47 (5.01, 6.10) | 4.85 (4.34, 5.57) | | 6.92 (6.34, 7.63) | 5.93 (5.63, 6.76) | 6.08 (5.26, 6.38) | 5.90 (5.04, 6.42) | 6.03 (5.10, 6.41) | 5.67 (5.07, 6.25) | 5.25 (4.68, 6.10) | 4.85 (3.87, 5.91) | 6.90 (6.54, 7.25) | | 5.88 (5.39, 6.53) | 5.98 (5.10, 6.17) | 5.53 (5.32, 5.76) | 5.24 (5.16, 5.31) | 4.89 (4.73, 4.94) | 4.52 (4.09, 5.01) | 4.11 (3.94, 4.90) | |
| Missing, n | 2 | | 3 | 3 | 3 | 3 | 2 | 2 | 3 | 3 | | 1 | 1 | 1 | 1 | 1 | 1 | 1 | | 2 | 3 | 2 | 4 | 3 | 3 | 2 | 2 | 1 | | 1 | 1 | 3 | 1 | 1 | 2 | 0 | |
| logTTP | 1.94 (1.90, 2.04) | | 2.04 (1.97, 2.11) | 2.03 (1.99, 2.13) | 2.09 (2.02, 2.20) | 2.09 (2.02, 2.22) | 2.12 (2.07, 2.21) | 2.14 (2.10, 2.21) | 2.16 (2.09, 2.21) | 1.93 (1.90, 2.04) | | 1.98 (1.96, 2.07) | 2.06 (2.01, 2.11) | 2.06 (2.02, 2.15) | 2.09 (2.03, 2.16) | 2.11 (2.06, 2.19) | 2.15 (2.10, 2.17) | 2.20 (2.13, 2.28) | | 1.96 (1.90, 2.06) | 2.07 (1.97, 2.21) | 2.07 (1.99, 2.21) | 2.07 (2.03, 2.24) | 2.16 (2.06, 2.27) | 2.19 (2.07, 2.28) | 2.20 (2.09, 2.32) | 2.18 (2.08, 2.33) | 1.94 (1.92, 2.02) | | 2.21 (2.15, 2.27) | 2.22 (2.20, 2.28) | 2.28 (2.25, 2.29) | 2.28 (2.28, 2.37) | 2.32 (2.29, 2.36) | 2.40 (2.35, 2.44) | 2.42 (2.29, 2.47) | |
| Missing, n | 0 | | 0 | 0 | 0 | 0 | 0 | 0 | 0 | 0 | | 0 | 0 | 0 | 0 | 0 | 0 | 0 | | 0 | 0 | 0 | 0 | 1 | 0 | 0 | 2 | 0 | | 0 | 0 | 0 | 0 | 0 | 0 | 0 | |
| logMBLA | 7.32 (6.60, 7.83) | | 6.67 (6.37, 7.46) | 6.80 (5.92, 7.29) | 6.56 (6.08, 7.10) | 6.22 (5.49, 7.05) | 6.42 (5.33, 6.81) | 6.07 (5.84, 6.45) | 5.93 (5.50, 6.45) | 7.53 (7.00, 7.82) | | 7.43 (7.21, 7.72) | 6.94 (6.69, 7.35) | 6.99 (6.54, 7.23) | 6.74 (6.48, 7.15) | 6.55 (6.02, 6.81) | 6.30 (5.76, 6.85) | 6.17 (5.75, 6.51) | | 7.43 (7.12, 7.65) | 7.19 (6.02, 7.57) | 7.05 (6.63, 7.55) | 7.02 (6.30, 7.43) | 6.54 (5.92, 7.26) | 6.29 (5.61, 6.82) | 6.42 (5.16, 6.90) | 6.61 (4.74, 7.16) | 7.45 (7.00, 7.81) | | 6.66 (6.17, 7.29) | 6.53 (5.93, 6.91) | 6.35 (5.88, 6.77) | 6.42 (6.13, 7.12) | 6.13 (5.83, 6.52) | 5.57 (5.30, 6.34) | 5.75 (4.71, 6.07) | |
| Missing, n | 0 | | 0 | 1 | 0 | 1 | 0 | 0 | 0 | 0 | | 0 | 0 | 0 | 0 | 0 | 0 | 0 | | 0 | 0 | 0 | 2 | 1 | 2 | 0 | 0 | 0 | | 1 | 1 | 1 | 0 | 0 | 0 | 0 | |
| ^1^Median (Q1, Q3). CFU, Colony Forming Units; MGIT TTP, Mycobacterial Growth Indicator Tube Time to Positivity; TB-MBLA, Tuberculosis Mycobacterial Load Assay; c/rxn, copies per reaction. | | | | | | | | | | | | | | | | | | | | | | | | | | | | | | | | | | | | |  |

**Table S2. Summary statistics by assay, by treatment arm for Phase IIa**.

**Table S3. CVs for the fixed effects from the dose-response mixed effect models.**

| **Treatment arm** | **CFU** | **TB-MBLA** | **MGIT TTP** | **PC1** | **CFU + MGIT TTP PC1** | **CFU+TB-MBLA PC1** | **TTP+TB-MBLA PC1** |
| --- | --- | --- | --- | --- | --- | --- | --- |
| **250 mg** | 20.2 | 18.5 | 16.6 | 16.8 | 17.3 | 17.6 | 16.5 |
| **500 mg** | 21.1 | 19.6 | 16.0 | 17.1 | 17.2 | 18.4 | 16.9 |
| **1000 mg** | 16.4 | 19.8 | 15.0 | 15.5 | 14.4 | 16.3 | 15.7 |
| **HRZE** | 14.0 | 16.9 | 9.2 | 11.5 | 10.5 | 13.7 | 11.7 |

CV, coefficient of variation. HRZE, isoniazid, rifampicin, pyrazinamide, and ethambutol standard of care treatment.

**Table S4. PK-PD model parameters for TB-MBLA.**

| **Parameter** | **Coefficient estimate (% RSE)** | **Interindividual variability [CV%] (% RSE)** |
| --- | --- | --- |
| Baseline bacterial load [log10 TB-MBLA c/rxn] | 7.14 (2%) | 10.7 (13%) |
| Slope 1 [log10 TB-MBLA c/rxn * day-1] | 0.224 (14%) |  |
| Slope 2 [log10 TB-MBLA c/rxn * day-1] | 0.0703 (15%) | 84.5 (14%) |
| Node [days] | 2(FIX) |  |
| Additive error [log10 TB-MBLA c/rxn -1] | 0.245 (23%) |  |
| Additive error replicates [log10 TB-MBLA c/rxn -1] | 0.00664 (42%) |  |

CV, coefficient of variation; RSE, relative standard error; c/rxn, copies per reaction.

**Table S5. PK-PD model parameters for PC1 (MGIT TTP + CFU + TB-MBLA).**

| **Parameter** | **Coefficient estimate (% RSE)** | **Interindividual variability [CV%] (% RSE)** |
| --- | --- | --- |
| Baseline PCA1 | -1.38 (12%) | 104 (19%) |
| Emax slope 1 PC1 [/day] | 0.617 (17%) |  |
| E50 | 5430 (59%) |  |
| Slope 2 PC1 [/day] | 0.109 (13%) | 66.7 (15%) |
| Node [days] | 2.88 FIX |  |
| Additive error PC1 | 0.437 (18%) |  |

PC1, First principal component; CV, coefficient of variation; RSE, relative standard error.

**Table S6. PK-PD model parameters for PCA1 (MGIT TTP + CFU).**

| **Parameter** | **Coefficient estimate (% RSE)** | **Interindividual variability [CV%] (% RSE)** |
| --- | --- | --- |
| Baseline PC1 | -1.20 (13%) | 98.9 (14%) |
| Emax slope 1 PC1 | 0.547 (22%) | 25.1 (58%) |
| E50 | 9470 (66%) |  |
| Slope 2 PC1 | 0.0816 (11%) | 60.8 (23%) |
| Node [days] | 2.88 FIX |  |
| Additive error PC1 | 0.216 (6%) |  |

PC1, First principal component; CV, coefficient of variation; RSE, relative standard error.

**Table S7. Summary of exposure-response effects for BTZ-043 using different output markers.**

| Bacteriological markers | | | Effect | ΔOFV (P-value) |
| --- | --- | --- | --- | --- |
| CFU | TTP | MBLA |  |  |
| X |  |  | BTZ-043_total_ AUC_0-24_ Emax | -11.30 (P=3.5x10^-3^) |
|  | X |  | BTZ-043_total_ AUC_0-24_ Emax | -11.11 (P=3.9x10^-3^) |
|  |  | X | BTZ-043_total_ AUC_0-24_ linear | 0 (NA) |
| X | X |  | BTZ-043_total_ AUC_0-24_ Emax | -12.55 (P=3.9x10^-4^) |
| X | X | X | BTZ-043_total_ AUC_0-24_ Emax | -5.535 (P=0.0186) |
|  | X | X | BTZ-043_total_ AUC_0-24_ Emax | -3.669 (P=0.0554) |
| X |  | X | BTZ-043_total_ AUC_0-24_ Emax | -3.479 (P=0.0622) |

The table presents the ΔOFV and corresponding P-values for exposure-response relationships analysed with MGIT TTP, CFU, TB-MBLA and all pairwise combinations derived via pPCA. The combined MGIT TTP-CFU marker demonstrates the greatest statistical resolution, emphasizing its potential to enhance analysis in early bactericidal activity (EBA) trials. Emax refers to the classic Emax parameterization of the exposure-response model. ΔOFV, change in objective function value; pPCA, probabilistic PCA; MGIT TTP, Mycobacterial Growth Indicator Tube Time To Positivity; CFU, colony forming units; TB-MBLA, Tuberculosis Mycobacterial load assay.

**Figure S1. Forest plot for fixed effect coefficients from linear mixed effect modelling for all iterations of PCA.**

The resulting coefficients from the fixed effect represent the average slope per treatment arm. For MGIT TTP and PC biomarkers, a positive slope represents bactericidal activity, with greater values representing greater effect. Conversely, for CFU and TB-MBLA markers, a negative slope indicates presence of bactericidal activity, with greater values indicating greater effect. CFU, colony forming units, MGIT TTP, Mycobacterial Growth Indicator Tube Time to Positivity; TB-MBLA, Tuberculosis Mycobacterial Load Assay

**Figure S2. Coefficient of variation values for fixed effect coefficients from linear mixed effect modelling for all iterations of PCA.**

CV values for each model coefficient. A smaller CV indicates greater precision of the model estimate. CFU, colony forming units, MGIT TTP, Mycobacterial Growth Indicator Tube Time to Positivity; TB-MBLA, Tuberculosis Mycobacterial Load Assay; CV, coefficient of variation.

**Figure S3: Spaghetti plots of per-patient trajectories in CFU, MGIT TTP and PC1 over time in the validation dataset.**

The coloured lines represent the median over time. Breaks in individual lines represent missing data, either from sample availability or bacterial contamination.
